# A Guideline for Guidelines: A Novel Method to Assess the Helpfulness of Medical Guidelines

**DOI:** 10.3390/jcm13133783

**Published:** 2024-06-27

**Authors:** Akos Koller, Johanna Takács

**Affiliations:** 1 Department of Physiology, New York Medical College, Valhalla, NY 10595, USA; 2 Department of Morphology and Physiology, Faculty of Health Sciences, Semmelweis University, H-1088 Budapest, Hungary; 3 Department of Translational Medicine, Faculty of Medicine, HUN-REN-SE Cerebrovascular and Neurocognitive Disease Research Group, Semmelweis University, H-1094 Budapest, Hungary; 4 Research Center for Sports Physiology, Hungarian University of Sports Science, H-1123 Budapest, Hungary; 5 Department of Social Sciences, Faculty of Health Sciences, Semmelweis University, H-1088 Budapest, Hungary; takacs.johanna@semmelweis.hu

**Keywords:** mathematical analysis, helpfulness, quality, certainty, uncertainty

## Abstract

**Background/Objectives**: The recommendations included in medical guidelines (GLs) provide important help to medical professionals for making clinical decisions regarding the diagnosis and treatment of various diseases. However, there are no systematic methods to measure the helpfulness of GLs. Thus, we developed an objective assessment of GLs which indicates their helpfulness and quality. We hypothesized that a simple mathematical analysis of ‘Recommendations’ and ‘Evidence’ would suffice. **Methods**: As a proof of concept, a mathematical analysis was conducted on the ‘2020 European Society of Cardiology Guidelines on Sports Cardiology and Exercise in Patients with Cardiovascular Disease Guideline’ (SCE-guideline). First, the frequencies of Classes of Recommendations (CLASS) and the Levels of Evidence (LEVEL) (n = 159) were analysed. Then, LEVEL areas under CLASS were calculated to form a certainty index (CI: −1 to +1). **Results**: The frequency of CLASS I (‘to do’) and CLASS III (‘not to do’) was relatively high in the SCE-guideline (52.2%). Yet, the most frequent LEVEL was C (41.2–83.8%), indicating only a relatively low quality of scientific evidence in the SCE-guideline. The SCE-guideline showed a relatively high CI (+0.57): 78.4% certainty and 21.6% uncertainty. **Conclusions**: The SCE-guideline provides substantial help in decision making through the recommendations (CLASS), while the supporting evidence (LEVEL) in most cases is of lower quality. This is what the newly introduced certainty index showed: a tool for ‘quality control’ which can identify specific areas within GLs, and can promote the future improvement of GLs. The newly developed mathematical analysis can be used as a Guideline for the Guidelines, facilitating the assessment and comparison of the helpfulness and quality of GLs.

## 1. Introduction

The first medical guideline (GL) may have been written by Hippocrates [1]. In the modern age, in 1992, the Institute of Medicine reported a GL to help physicians and patients make decisions in particular disease conditions [2]. Since then, numerous GLs have been published. Indeed, currently, medical societies publish several GLs to help medical professionals make decisions in specific health conditions. The advice provided by these GLs significantly aids in making clinical decisions regarding the diagnosis and treatment of various diseases [3,4].

GLs are based on the Classes of Recommendations (CLASS) and Levels of Evidence (LEVEL). There are no systematic methods to measure the helpfulness or quality of GLs, i.e., how much help they provide [5,6]. Over the past decade, several studies have pointed out that only a small percentage (<15%) of the recommendations in clinical practice GLs for the management of cardiovascular diseases are based on Level A evidence [7,8,9,10,11], i.e., there is a lack of the highest level of evidence from multiple randomized clinical trials and meta-analyses. Moreover, some of these studies have revealed a consistent time trend or slight improvements in this level of evidence over almost a decade [7,9,10,11]. There is a need to further develop methods to assess GLs, which can be improved by including implementation advice and translating evidence into recommendations [12,13]. 

Thus, it is important to develop a simple mathematical analysis to objectively assess the help provided by GLs in making informed decisions in various health conditions. We hypothesized that a simple mathematical analysis of ‘Recommendations’ and ‘Evidence’ would suffice. To test this hypothesis, we performed a mathematical analysis of the ‘2020 European Society of Cardiology Guidelines on Sports Cardiology and Exercise in Patients with Cardiovascular Disease Guideline’ (SCE-guideline) [14].

## 2. Materials and Methods

In the present study, no patient data were included. The SCE-guideline was analysed based on the frequency of the Classes of Recommendations (CLASS) and the Levels of Evidence (LEVEL) in the SCE-guideline. The recommendations have three classes: I (is recommended or is indicated), IIa,b (should or may be considered), and III (is not recommended). The evidence has three LEVELs: A (multiple randomized clinical trials, meta-analyses), B (single randomized clinical trials, large non-randomized studies), and C (opinion of experts, small studies, retrospective studies, registries).

We examined (1) recommendations in the entire SCE-guideline and the two main parts of the SCE-guideline, which are (2) recommendations in ‘individuals with cardiovascular risk factors and ageing’ (RFA), and (3) in ‘clinical settings’ (CS), in line with the structure of the SCE-guideline. For the number of recommendations in SCE-guideline with the Classes of Recommendations and the Levels of Evidence, see Supplementary Table S1.

### Data Analysis

**Figure 1:**
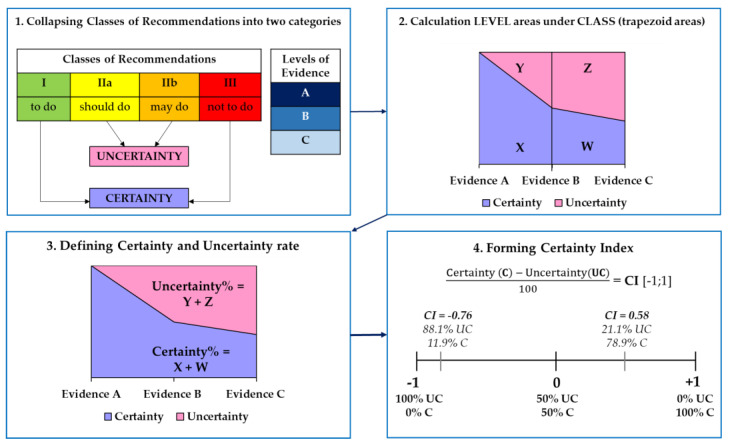
Illustration of the certainty Index. 1. Collapsing Classes of Recommendations into two categories: to define certainty and uncertainty, we collapsed the Classes of Recommendations into two categories: uncertainty (‘should + may do’) and certainty (‘to do + not to do’). 2. Calculation LEVEL areas under CLASS: we calculated the areas using the trapezoid area formula: $=\left(a+b\right)\frac{h}{2}$, where ‘*a*’ and ‘*b*’ are the bases (parallel sides) and ‘*h*’ is the height of the trapezoid. 3. Defining certainty and uncertainty rate: Based on the calculation in step 2, we expressed trapezoid areas in percentages. 4. Forming certainty index: Based on the percentages in step 3, an index of certainty/uncertainty was formed which ranges from −1 to +1; a value of −1 implies 100% uncertainty, a value of +1 implies 100% certainty, and a value of 0 implies fifty–fifty certainty and uncertainty. The depicted certainty indexes (CI = −0.76 and CI = +0.58) are random examples showing the meaning of a certainty index with the rate of the percentages of certainty and uncertainty.

The mathematical analysis of the SCE-guideline was based on our previous study [15], which was further extended by introducing the certainty index. Descriptive statistics were used to summarise the frequency of CLASS and LEVEL. Frequency distributions of LEVEL by CLASS were applied to examine the differences between Evidence A, B, and C on CLASS. To define certainty and uncertainty, Classes of Recommendations were collapsed into two categories: (1) uncertainty (‘should or may do’) and (2) certainty (‘to do or not to do’). Subsequently, we calculated LEVEL areas under CLASS using the trapezoid area formula: $=\left(a+b\right)\frac{h}{2}$, where ‘*a*’ and ‘*b*’ represent the bases (parallel sides) and ‘*h*’ is the height of the trapezoid. Finally, we formulated an index of certainty/uncertainty (ranging from −1 to +1; a value of −1 implies 100% uncertainty, a value of +1 implies 100% certainty, and a value of 0 denotes fifty–fifty certainty and uncertainty (Figure 1). Data analyses were conducted using Excel 2016 (v16.0) and IBM SPSS Statistics for Windows, Version 25.0 (IBM Corp. Released 2017. Armonk, NY, USA: IBM Corp.).

## 3. Results

A total of 159 CLASS and LEVEL data points were examined in the SCE-guideline, and the two main parts of the SCE-guideline were ‘individuals with cardiovascular risk factors and older adults’ (RFA) (n = 17) and ‘clinical settings’ (CS) (n = 142). 

### 3.1. Frequency Distribution of CLASS and LEVEL in the SCE-Guideline

#### 3.1.1. Classes of Recommendations (CLASS I, IIa, IIb, III)

The frequency of CLASS I (recommended/indicated) and CLASS III (not recommended) was relatively high in both the SCE-guideline (52.2%) and the CS part (50.7%). The RFA part showed the highest percentage in these Classes of Recommendations (64.7%). This indicated that the percentages for the ‘intermediate’ categories (should do—CLASS IIa; may do—CLASS IIb) were also relatively high (ranging from 35.3% to 49.3%), indicating a slightly low strength of the recommendations (Table 1).

#### 3.1.2. Levels of Evidence (Evidence A, B, C)

The percentage of Evidence A was below 50% for the RFA part (35.3%), and it was below 10% in the SCE-guideline (7.6%) as well as in the CS part (4.2%). We found that Evidence C was the most frequent (41.2–83.8%), indicating only relatively low quality of the scientific evidence in the SCE-guideline (Table 1).

### 3.2. Frequency Distribution of LEVEL by CLASS in the SCE-Guideline

**Figure 2:**
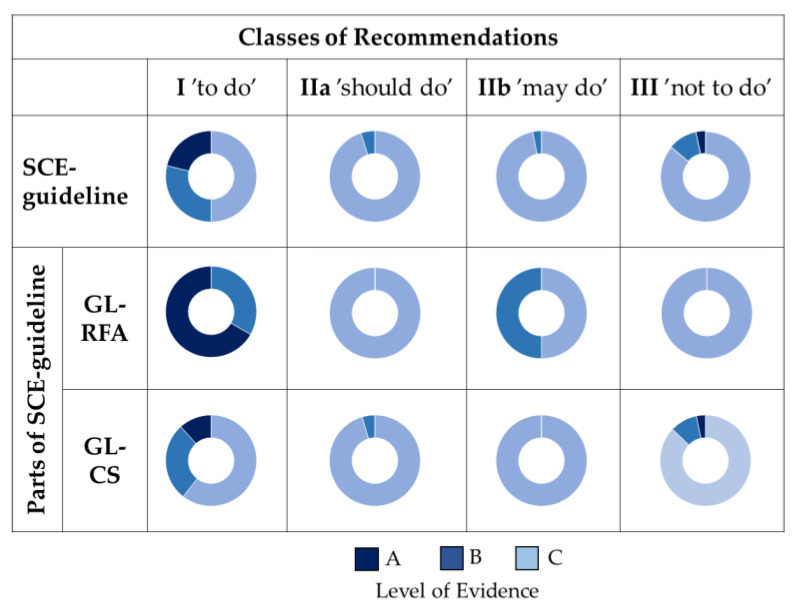
Visualization of the frequency distribution of LEVEL by CLASS in the SCE-guideline and the two main parts (RFA: risk factors and ageing, CS: clinical settings, colour code is based on the SCE-guideline).

In the SCE-guideline, based on the frequency distribution of LEVEL by CLASS, the percentage of Evidence A was low in each Class of Recommendations. The percentage of Evidence A was only 21.2% in ‘to do’ Class I. The RFA part showed the highest percentage of Evidence A in ‘to do’ Class I (66.7%). In contrast, in the CS part, the percentage of Evidence C was the highest in each class of recommendations (60.5–100%) (Figure 2).

### 3.3. Certainty Index in the SCE-Guideline

**Figure 3:**
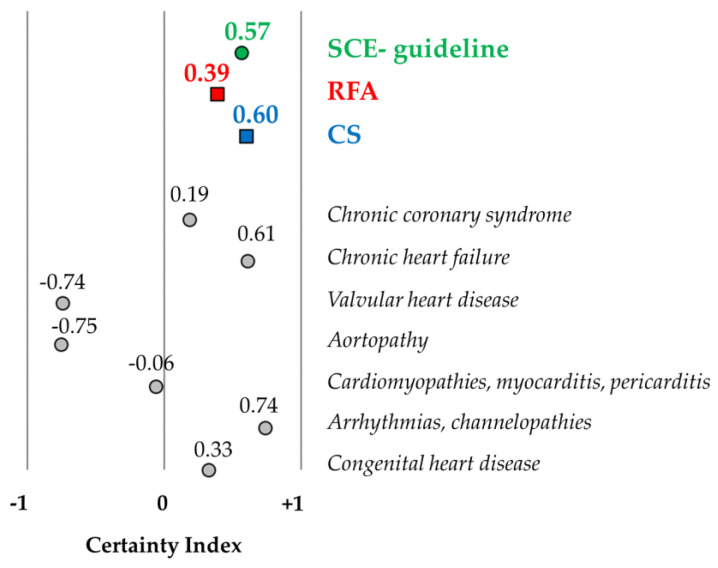
Certainty Index in the SCE-guideline and the two main parts of the SCE-guideline (RFA: ‘Risk factors and Ageing’ and CS: ‘Clinical settings’), as well as for patients with certain cardiovascular diseases included in the RFA part. Certainty index (CI), −1: 100% uncertainty, +1: 100% certainty, 0: 50% uncertainty, and 50% certainty. The SCE-guideline showed a CI of 0.57, which means 78.4% certainty and 21.6% uncertainty. The RFA part showed a smaller CI (0.39: 69.7% certainty and 30.3% uncertainty). In the CS part, the CI was somewhat higher, with 0.60 (79.8% certainty and 20.2% uncertainty). Certainty indexes for patients with certain cardiovascular diseases included in the RFA part are in italics. Negative CIs mean higher rates of uncertainty; for example, valvular heart disease showed a CI of −0.74, which means 87.1% uncertainty and 12.9% certainty.

The certainty indexes were 0.57 and 0.60 in the SCE-guideline and CS part, respectively, indicating a relatively high rate of certainty. The value was much lower for the RFA part (CI: 0.39). The certainty index is depicted in Figure 3 in the SCE-guideline and the two main parts (RFA and CS), as well as for patients with specific cardiovascular diseases, who are included in the RFA part.

## 4. Discussion

The present study introduced a mathematical analysis to objectively assess the effectiveness (helpfulness) of GLs, which will facilitate the comparison and future developments of GLs. Overall, the analysis revealed that the SCE-guideline provides helpful recommendations regarding exercise/sports activities on CVD, while the supporting evidence, in most cases, is of lower quality.

In the present study, no opinions were given regarding the scientific content of the SCE-guideline. At the same time, the findings presented here can be compared with those revealed by our previous analytic study on several GLs, which showed that various GLs provide different strengths of recommendations and different qualities of evidence [15]. The higher levels of scientific evidence and/or the clinical significance of lower scientific evidence may be increased by conducting new basic science through experimental and clinical studies based on the revealed gaps in the GLs; hence, helpfulness and quality may be increased in clinical decision making. Thus, the introduced mathematical analysis can also be used to compare the helpfulness and quality of GLs between different subfields within the same medical field or across broader medical areas, such as cardiology, neurology, etc. 

The SCE-guideline presented ‘strong’ recommendations, albeit primarily supported by lower-quality evidence. In various disease conditions, both the strength of recommendations and the levels of evidence varied. Previous studies have evaluated GLs by examining the frequencies of Classes of Recommendations and/or Levels of Evidence, either independently or dependently between each other, without providing meaningful explanations for these findings [7,8,9,10,11]. Assessing the helpfulness and quality of GLs using certainty/uncertainty (certainty index) may be more practical, as it can identify specific areas within GLs that require improvement and promote future enhancements.

### 4.1. The Certainty Index

To better elucidate the helpfulness and quality of GLs in terms of certainty and uncertainty rates, we introduced the certainty index (CI), which ranges from −1 to +1. This allows for the comparison of various GLs and the identification of specific areas that require investigation in future studies. It is also recommended to assess the certainty index concerning the type and actions of recommendations [16]. An important consideration is that it is difficult to include all risk factors (such as obesity, dyslipidaemia, hypertension, diabetes, etc.) and different pathomechanisms within a single GL. Additionally, assigning specific values for the type, intensity, duration, and frequency of exercise as well as various cardiovascular conditions is complex. Achievable, inter-individual differences such as age, gender, or co-morbidities [17,18] hinder the grouping of patients into specific categories. 

### 4.2. Bias in GLs Such as Sex, Age, and Other Factors

It is important to recognize that personalized exercise and sports therapy recommendations can be challenging due to various factors. For instance, previous trials have often included a limited number of women, despite recognized sex-related differences in cardiovascular disease mechanisms, presentations, diagnosis, and treatment [19]. Consequently, the sex-specific response to exercise and therapeutic use of exercise modalities remain incompletely understood [20,21,22]. Moreover, age-related variations in cardiovascular parameters are often based on idealized young male models rather than solid evidence, given the underrepresentation of older patients and a higher number of ‘best case scenarios’ in RCTs [23]. In addition, in conditions with diagnostic grey areas, such as heart failure [24] or extreme environmental conditions like high altitude [25] or temperature changes, assigning personalized exercise regimens is challenging.

In general, it is known that decisions have some bias, as has been revealed by human experimental studies [26] showing that cognitive bias always occurs in decision making. Therefore, cognitive bias must also be considered in the preparation of GLs, as the authors may have varying levels of scientific expertise and clinical experience, potentially leading to unrecognized conflicts of interest.

### 4.3. Future Considerations

Due to methodological and ethical constraints, Evidence A cannot always be provided in human studies. Therefore, it may be suggested that the certainty of a GL cannot be or should not be increased by only Evidence A, but lower-quality evidence can be accepted as sufficient in certain cases. Thus, in such cases, it may be advisable to give greater weight to lower-quality evidence, thereby avoiding the limitations of rigid classifications based on dichotomized or categorised systems such as the GRADE framework, AAP, or ESC Guidelines Classifications Scheme in the current guideline [27,28,29]. 

While various methods have been proposed for grading recommendation strength, developers generally agree that determining the strength of action differs from rating the overall quality of evidence. One can argue that high-quality evidence (such as grade A) does not always warrant strong recommendations. Conversely, recommendations, including strong ones, may be feasible even with lower-quality evidence (such as grades B, C, or X) [30]. The primary factor modifying this is the benefit–harm assessment, as defined in the preceding section on action statement profiles.

The method for determining the strength of recommendations developed by the American Academy of Paediatrics (AAP) is simple, transparent, and clinically relevant [27]. Similar to the GRADE approach [20], it considers the aggregate evidence level and benefit-harm assessment as primary rating determinants. However, GRADE offers only two levels of action strength (‘strong recommendation’ and ‘weak recommendation’), whereas AAP provides three levels (‘strong recommendation’, ‘recommendation’, and ‘option’). Empirical experience in developing GLs suggests that three levels support more flexible decision making and are better accepted by clinicians [31].

### 4.4. Limitations of the Study

There are potential limitations in this analytic study. There are inherent ‘potential limitations and harms of guidelines’ [12], such as the fact that the recommendations may be wrong for a group of or at least one individual patient, and errors which were carried forward into our analysis. Finally, it is important to note that no prior analytic research of this kind on the GLs existed. Therefore, it is likely that the methodology applied in the current study will be refined and improved in future research.

It is of note that a high-quality, evidence-based guideline does not necessarily guarantee that the recommendations will be applied in healthcare practice [32,33]. Various factors can act as major barriers to GL adherence, including the complexity of GLs, a high number of ‘should be/may be’ recommendations, and time constraints due to clinical responsibilities [34,35,36]. Recommendations can be either strong or conditional; however, it is important to highlight the meaning of conditional recommendations. The intended meaning is that most patients/physicians would choose the recommended action, but a substantial number of patients/physicians would not [37]. Implementation science could significantly contribute to the development of clinical practice GLs, particularly in promoting adherence to recommendations lacking high-level evidence [38].

## 5. Conclusions

The 2020 ESC SCE-guidelines provides substantial help in decision making through the recommendations (CLASS), while the supporting evidence (LEVEL), in most cases, is of lower quality. This is what the newly introduced certainty index showed. Our analysis provides a tool for ‘quality control’ with the newly introduced certainty index, and by identifying specific areas within a certain GL, it can promote the future improvement of GLs. The newly developed mathematical analysis can be used as a Guideline for Guidelines, facilitating the assessment and comparison of the helpfulness and quality of GLs.

## Figures and Tables

**Table 1:** Frequency distribution of Classes of Recommendations and Levels of Evidence in the SCE-guideline and the two main parts (RFA: risk factors and ageing, CS: clinical settings; colour code is based on the SCE-guideline).

**Classes of Recommendations**	**SCE-Guideline**	**Parts of SCE-Guideline**	
		**RFA**	**CS**
**Class I**—Is recommended/ is indicated	32.7%	52.9%	30.3%
**Class IIa**—Should be considered	27.7%	23.5%	28.2%
**Class IIb**—May be considered	20.1%	11.8%	21.1%
**Class III**—Is not recommended	19.5%	11.8%	20.4%
**Levels of Evidence**	**SCE-Guideline**	**Parts of SCE-Guideline**	
		**RFA**	**CS**
**Evidence A**—multiple randomized clinical trials or meta-analysis	7.6%	35.3%	4.2%
**Evidence B**—single randomized clinical trials or large non-randomized studies	13.2%	23.5%	12.0%
**Evidence C**—experts and/or small studies, retrospective studies, registries	79.2%	41.2%	83.8%

## Data Availability

The datasets used during the current study are available from the corresponding author upon reasonable request.

## Supplementary Materials

The following supporting information can be downloaded at: https://www.mdpi.com/article/10.3390/jcm13133783/s1, Table S1: The number of Classes of Recommendations and Levels of Evidence in the ‘2020 European Society of Cardiology Guidelines on Sports Cardiology and Exercise in Patients with Cardiovascular Disease Guideline’ (SCE-guideline), and in the two main parts of the SCE-guideline: ‘in individuals with cardiovascular risk factors and ageing’ (RFA) and ‘in clinical settings’ (CS).
