# Supplementary material for: A Guideline for Guidelines: A Novel Method to Assess the Helpfulness of Medical Guidelines
Source: J Clin Med. 2024 Jun 27;13(13):3783. doi: 10.3390/jcm13133783 (PMC11242354; doi:10.3390/jcm13133783)
Supplement: Supplementary file 1 [file jcm-13-03783-s001.zip › jcm-2998731-supplementary.pdf]

Supplementary Table S1. The number of Classes of Recommendations and Levels of Evidence in the '2020 European Society of Cardiology Guidelines on Sports Cardiology and Exercise in Patients with Cardiovascular Disease Guideline' (SCE-guideline), and in the two main parts of the SCE-guideline: 'in individuals with cardiovascular risk factors and ageing' (RFA) and 'in clinical settings' (CS)

**SCE-guideline (N=159)**	**Levels of Evidence**		
	**A (n=12)**	**B (n=21)**	**C (n=126)**
**Classes of Recommendations**			
**I 'to do' (n=52)**	11	15	26
**IIa 'should do' (n=44)**	0	2	42
**IIb 'may do' (n=32)**	0	1	31
**III 'not to do' (n=31)**	1	3	27

**RFA (n=17)**	**Levels of Evidence**		
	**A (n=6)**	**B (n=4)**	**C (n=7)**
**Classes of Recommendations**			
**I 'to do' (n=9)**	6	3	0
**IIa 'should do' (n=4)**	0	0	4
**IIb 'may do' (n=2)**	0	1	1
**III 'not to do' (n=2)**	0	0	2

**CS (n=142)**	**Levels of Evidence**		
	**A (n=6)**	**B (n=17)**	**C (n=119)**
**Classes of Recommendations**			
**I 'to do' (n=43)**	5	12	26
**IIa 'should do' (n=40)**	0	2	38
**IIb 'may do' (n=30)**	0	0	30
**III 'not to do' (n=29)**	1	3	25

Notes: Classes of Recommendations: I (is recommended or is indicated), IIa,b (should or may be considered), III (is not recommended); Levels of Evidence: A (multiple randomized clinical trials, meta-analyses), B (single randomized clinical trials, large non-randomized studies) and C (opinion of experts, small studies, retrospective studies, registries).
